# 1272. Teaching the ABCs of ASB in a 60-minute educational intervention. A quality improvement project.

**DOI:** 10.1093/ofid/ofad500.1112

**Published:** 2023-11-27

**Authors:** Paulina Vega Enriquez, Ruayda Bouls, Ed W Alvarado, Jose Campo Maldonado

**Affiliations:** University of Washington , Seattle, Washington; University of Texas Rio Grande Valley School of Medicine, Harlingen, Texas; University of Texas Rio Grande Valley School of Medicine, Harlingen, Texas; University of Texas Rio Grande Valley School of Medicine, Harlingen, Texas

## Abstract

**Background:**

Asymptomatic bacteriuria (ASB) refers to the presence of bacteria in the urine of individuals that do not present with symptoms of urinary tract infection (UTI). Treatment for ASB is not recommended in most cases. ^[1]^ Previous studies have shown that provider education on proper antibiotic use has been effective in mitigating the ongoing wave of antimicrobial-resistant organisms across hospitals in the United States.^[2]^ We aimed to decrease the proportion of cases classified as having asymptomatic bacteria by 20% in four months after the intervention using a small group discussion and a presentation.

**Methods:**

We evaluated the rates of inappropriate antimicrobial prescribing for asymptomatic bacteriuria (ASB) for 4 months before educational intervention and 4 months after. Data was extracted from the electronic medical record (Cerner®), imported into REDCap®, and with standardized manual chart review. Data analysis was completed using STATA® 17 & 18.

**Results:**

The mean duration of antibiotic therapy was 7.9 days in the pre-intervention group and 6.1 days in the post-intervention group. See Table 1 for more details on demographics. Following the intervention, the rate of inappropriate use of antimicrobial therapy in ASB was reduced by 29.8% (p-value 0.035). The mean duration of antibiotic therapy decreased by almost 2 days in the post-intervention group.

**Figure:**
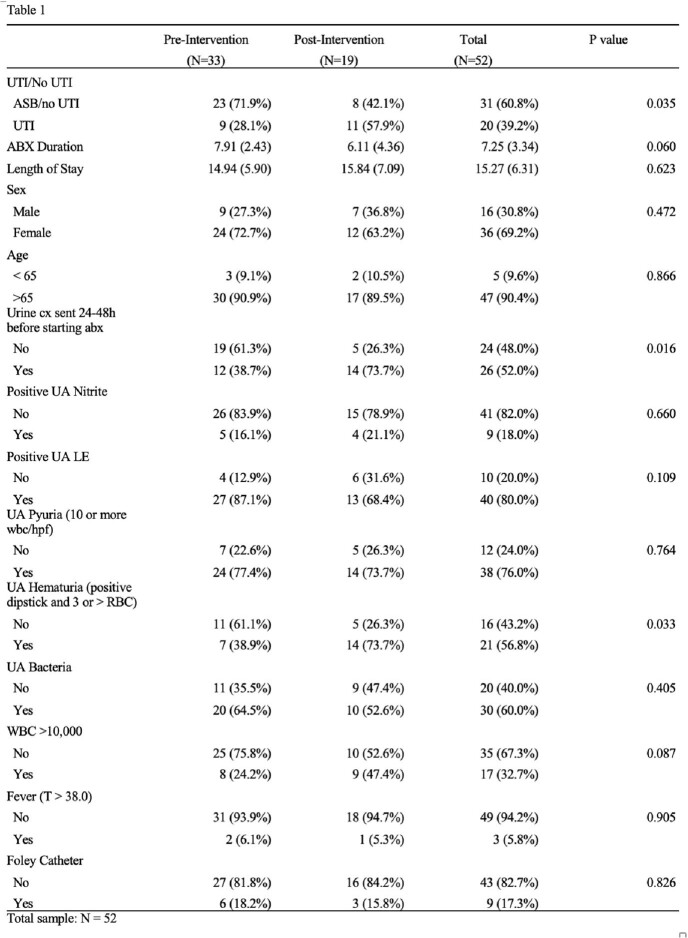

**Conclusion:**

This quality improvement project suggests that focused interventions of short interactive educational sessions in small groups may reduce the inappropriate use of antibiotics in ASB by almost 30%. The mean duration in days of antibiotic treatment decreased from 7.9 days to 6.1 days in the pre and post-intervention groups (p-value 0.060). Of note the total number of cases with providers selecting UTI as an indication was also lower in the post-intervention group and although we can’t determine causality, it provided evidence of a lower number of cases, providers initially classified as UTI between the pre and post-intervention period from 33 to 19. Further research may include incorporating small groups and interprofessional educational interventions to help implement good antimicrobial stewardship in inpatient care facilities. ^[4,5,6]^

**Disclosures:**

**All Authors**: No reported disclosures

